# Tamponnade péricardique révélant un lupus érythémateux systémique au cours d'une leishmaniose viscérale atypique

**DOI:** 10.11604/pamj.2014.18.323.4344

**Published:** 2014-08-21

**Authors:** Hajer Ben Brahim, Maha Ben Nasr, Hela Boussaid, Ikbel Kooli, Abir Aouam, Adnene Toumi, Chawki Loussaief, Mohamed Chakroun

**Affiliations:** 1 Service des Maladies Infectieuses, CHU Fattouma, Bourguiba Monastir, Tunisie

**Keywords:** Leishmaniose viscérale, lupus érythémateux systémique, association, corticothérapie, visceral leishmaniasis, systemic lupus erythematosus, association, corticotherapy

## Abstract

L'association de leishmaniose viscérale (LV) et lupus érythémateux systémique (LES) est rare et grave. La similitude des signes cliniques peut être responsables de difficultés diagnostiques surtout quand les deux pathologies se déclarent de façon concomitante. Nous rapportons l'observation d'une malade qui avait présenté une association grave et atypique de LV et LES qui se sont déclarés en même temps. La LV était atypique par l'absence du syndrome tumoral, de la fièvre et la négativité de la sérologie leishmaniose. Le LES jusque là méconnu était révélé par une poussée sévère avec différentes atteintes viscérales. L’évolution était favorable sous amphotéricine-B et corticothérapie. En cas d'association des deux pathologies une surveillance particulière est nécessaire à fin de diagnostiquer à temps une atteinte viscérale grave du LES et démarrer précocement une prise en charge étiologique et symptomatique qui est le seul garant de l’évolution favorable.

## Introduction

La leishmaniose viscérale (LV) est une infection opportuniste grave du système réticulo-histiocytaire réalisant un tableau clinique qui ressemble au lupus érythémateux systémique (LES). Ainsi, chez un même patient la co-existence, de façon inaugurale, des deux pathologies peut entraîner un retard diagnostic de l'une ou de l'autre et aggraver le pronostic [1]. A travers cette observation nous rapportons les particularités d'une telle association.

## Patient et observation

Madame M. A âgée de 32 ans, sans antécédents pathologiques notables, était admise pour une altération de l’état général, arthralgies inflammatoires avec bicytopénie, sans fièvre associée, évoluant depuis 3 mois. L'examen à l'admission avait noté une pâleur cutanéo-muqueuse, absence d'hépato-splénomégalie et d'adénopathies palpables. L'auscultation cardio-pulmonaire était normale. L'examen ostéo-articulaire n'avait pas objectivé d'arthrites. L'hémogramme avait révélé une anémie normochrome normocytaire régénérative à 4,9 g/dl, une leucopénie à 2900 éléments/mm3, une lymphopénie à 800 éléments/mm3 et une thrombopénie à 240000 éléments/mm3. Le test de coombs direct était positif. L’électrophorèse des protides sériques avait montré une augmentation polyclonale des gammaglobulines à 42,9 g/l, une hypoalbuminémie à 22 g/l, et une hyperprotidémie à75 g/l. La créatinémie était normale et la protéinurie de 24 heures était négative. La vitesse de sédimentation était accélérée à 40 mm à la première heure et la CRP était normale à 5 mg/l. La ponction sternale avait montré une moelle riche avec nombreuses images d'hémophagocytose. La PCR leishmaniose, sur sang médullaire, était positive mais la sérologie leishmaniose en ELISA et western blot était négative. Le bilan immunologique avait montré la présence d'anticorps antinucléaires (AAN) à un titre 1/200. Les anticorps anti-DNA native, anti-Sm et anti-nucléosomes étaient tous positifs avec une hypocomplémentémie.

Le diagnostic de leishmaniose viscérale était retenu et la patiente était traitée par Glucantime (3 gr/j) arrêté à j3 de traitement devant l'apparition de signes de stibio-intolérance (toux, fièvre et vomissements) et stibio-intoxication (Insuffisance rénale et pancréatite). Elle était mise alors sous amphotéricine-B (Fungizone) à une dose progressive jusqu’à 1mg/Kg/j. À une dose cumulée de 320 mg d'amphotéricine-B, la patiente avait présenté une fièvre à 40°, une dyspnée et une douleur thoracique en rapport avec une polysérite à type de tamponnade péricardique et pleurésie bilatérale de grande abondance. Un drainage thoracique en urgence était réalisé ramenant un liquide exsudatif lymphocytaire dont la recherche d'AAN était positive. Le lendemain la patiente avait présenté une baisse brutale de l'acuité visuelle en rapport avec des foyers de choriorétinite. Le diagnostic de LES était retenu. La patiente avait bénéficiée de 3 bolus de solumedrol (1g/bolus/j), puis elle était mise sous prédnisone (1mg/Kg/j) pendant 6 semaines. L’évolution ultérieure sous corticothérapie était favorable avec un assèchement péricardique et pleural et amélioration des lésions oculaires. L'amphotéricine-B était arrêtée après une dose cumulée de 1gr avec un hémogramme normal. Après un recul de 6 mois la malade va bien avec une acuité visuelle normale. La sérologie et la PCR leishmaniose sont négatives.

## Discussion

Bien que la LV est une infection opportuniste favorisée par la dépression de l'immunité cellulaire et le traitement par les immunosuppresseurs, elle est rarement décrite en cas de LES. La prévalence de cette association chez les lupiques est estimée à 6% [1]. Dans les différentes associations décrites de LES et LV, L'atteinte féminine était prédominante (9F/ < 2H). L’âge moyen était de 29,7 ans (19-44). Les deux pathologies sont découvertes de façon concomitante dans 30% des cas [2]. Dans ce cas, la similitude des tableaux cliniques rend le diagnostic précoce difficile. La production d'auto-anticorps tels que les AAN, anti-ADN natifs et anti-phospholipides a été décrite dans la LV depuis 30 ans et expliquée par une activation polyclonale des cellules B et une réactivité croisée entre les antigènes Leishmaniens et humains [3]. Pour cette raison, le diagnostic de LES n’était pas retenu au début chez notre malade. En effet, on n'avait que trois critères de l'Americain College of Rheumatology (ACR) à savoir la lymphopénie et la positivité des AAN et anti-DNA natifs. La survenue de LV s'accompagne d'une poussée lupique dans 80% des cas [2–4]. Dans notre observation, c'est cette poussée sous forme de tamponnade, pleurésie et atteinte oculaire qui avait révélé le LES jusque la méconnu. La tamponnade est une complication rare, redoutable et de mauvais pronostic, retrouvée dans 2,5% des cas dans une cohorte de 395 lupiques et exceptionnellement révélatrice du LES. Sa survenue chez notre patiente était favorisée par la survenue concomitante de LV qui a aggravé la poussée lupique.

De l'autre coté, la présentation clinique de la LV au cours du LES est souvent atypique expliquant un retard diagnostique dans la plupart des cas. La fièvre prolongée était rapportée dans 63% cas, les adénopathies dans 50-70% des cas, l'hépato-splénomégalie dans 80% cas et l'atteinte rénale dans 15% de cas [1, 5]. Les anomalies hématologiques classiques au cours de la LV tel que l'anémie, la leucopénie et la thrombopénie peuvent être confondues avec les anomalies hématologiques classiques du LES [6]. La sérologie est souvent mise en défaut en cas d'association LES et LV, alors qu′elle constitue un argument biologique majeur dans la LV des sujets immunocompétents [7]. La négativité de la sérologie par immunofluorescence indirecte est due à la présence d'anticorps bloquants qui inhibent la liaison anticorps anti-leishmanies et l'antigène. Cependant, la technique ELISA, comparativement, montre une plus grande sensibilité. La technique la plus performante est le Western-blot. Elle permet la détection d′anticorps hautement spécifiques, notamment dirigés contre les antigènes 14kD et 16 kD [7, 8]. Dans notre cas, aussi bien l'ELISA et le Western-blot étaient négatifs. Le diagnostic formel de LV repose sur la mise en évidence du parasite dans un prélèvement pathologique. La ponction sternale offre la meilleure sensibilité (90%). La biologie moléculaire (PCR), détectant I′ADN parasitaire, est un outil principal dans le diagnostic des LV notamment chez les immunodéprimés [9]. Dés que le diagnostic de LV est retenu, le traitement doit être débuté en urgence. En Tunisie, la glucantine est le traitement de première ligne de la LV [10].

Dans les séries tunisiennes, la mortalité en cas d'association LV et LES est élevée, de l'ordre de 80%. Les principales causes de mortalités étaient le choc septique et la tamponnade dans respectivement 75 et 25% des cas [2].ce résultat ne rejoigne pas la littérature où la survenue de LV chez les lupiques ne compromet pas le pronostic vital et l’évolution sous traitement est favorable chez tous les patients traités au prix des rechutes précoces à un an du traitement en cas de lymphopénie sévère [1, 5, 6].

## Conclusion

La LV doit être recherchée systématiquement chez les patients suivis pour LES particulièrement devant une exacerbation d'une poussée évolutive, une fièvre inexpliquée au long cours et une cytopénie. En cas d'association des deux pathologies une surveillance particulière est nécessaire à fin de diagnostiquer à temps une atteinte viscérale grave du LES et démarrer précocement une prise en charge étiologique et symptomatique qui est le seul garant de l’évolution favorable.
